# Fetal large tubular oesophageal duplication cyst: A case report

**DOI:** 10.1002/ajum.12387

**Published:** 2024-05-28

**Authors:** Călina Maier, Radu Vlădăreanu, Raluca Tocariu, Marcela Șerban, Maria Olincă, Elvira Brătilă

**Affiliations:** ^1^ Department of Obstetrics and Gynecology Clinical Hospital of Obstetrics and Gynecology “Professor Doctor Panait Sîrbu” Giulesti Street No. 5, District 6 050786 Bucharest Romania; ^2^ “Carol Davila” University of Medicine and Pharmacy Eroii Sanitari Boulevard No. 8, District 5 050474 Bucharest Romania; ^3^ Department of Obstetrics and Gynecology Elias Emergency University Hospital Marasti Boulevard No. 17, District 1 011461 Bucharest Romania; ^4^ Department of Neonatology Clinical Hospital of Obstetrics and Gynecology “Professor Doctor Panait Sîrbu” Giulesti Street No. 5, District 6 050786 Bucharest Romania; ^5^ Department of Pathology, Clinical Hospital of Obstetrics and Gynecology “Professor Doctor Panait Sîrbu” Giulesti Street No. 5, District 6 050786 Bucharest Romania

**Keywords:** case report, esophageal duplication cyst, prenatal diagnosis

## Abstract

**Introduction:**

Oesophageal duplication cyst (EDC) is a rare congenital anomaly representing, after neuronal tumours, the second most common cause of posterior mass in children, with a prevalence of approximately 1/22,500 live births. Cervical cysts are very rare, and their antenatal detection is fairly uncommon.

**Methods:**

We report the case of an isolated large mediastinal and cervical tubular EDC diagnosed prenatally in the third trimester.

**Results:**

After birth, the baby became symptomatic developing respiratory distress due to the enlargement of the cyst and she underwent excision of the mass. The post‐operative evolution was very good.

**Conclusion:**

Our purpose was to raise awareness of the ultrasonographic features of this condition, thus improving the rate of prenatal diagnosis and offering the parents a proper counselling regarding the prognosis and the need for a further multidisciplinary approach after birth.

## Introduction

Oesophageal duplication cysts (ODC) are the second most common duplication cysts following small bowel duplication cysts, accounting for approximately 10–15% of gastrointestinal duplication cysts.^1^ They are considered rare congenital cystic malformations that appear secondary to a faulty intrauterine recanalisation of the oesophagus during the 4–8th weeks of development.^2^ ODCs tend to have a male predominance (2:1) and are usually diagnosed (up to 80%) during infancy, as only a few patients remain asymptomatic until adulthood.^3^ In newborns, the most common presenting symptoms are respiratory distress secondary to airway compression, dysphagia and stridor,^4^ which is consistent with our case.

Most ODCs are likely to be localised in the lower third of the oesophagus; involvement of the cervical region is distinctly rare.^5^ Although intrathoracic, cervical and intraabdominal ODCs have been detected prenatally before,^6^ we present a case which is noteworthy for involving intrauterine ascertainment of mediastinal and cervical location of an isolated large ODC.

## Case report

**Figure 1:**
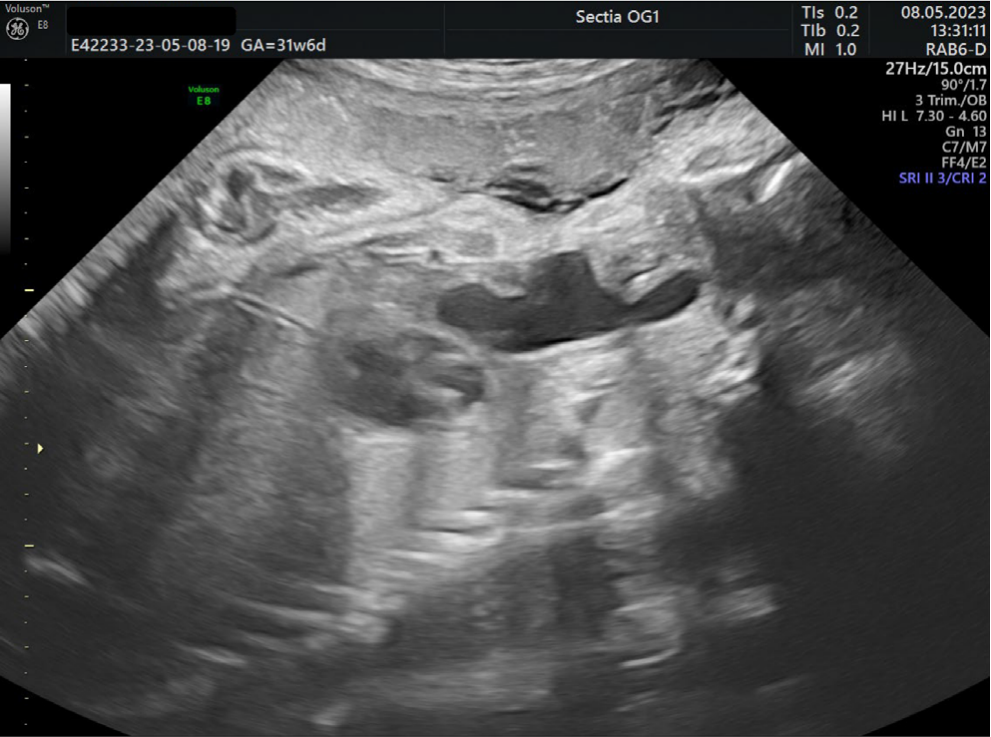
Esophageal duplication cyst at 31 weeks 6 days of pregnancy. A sagittal view of the fetal neck and thorax showing a cystic mass with irregular shape situated in the mediastinum and with cervical extension.

We present the case of a 23‐year‐old primigravida with an unremarkable medical history who was referred for the third‐trimester anomaly scan in our Fetal‐Maternal Medicine Department at 32 weeks of gestation. The non‐invasive prenatal testing performed in the first trimester revealed a low risk for trisomies 21, 18 and 13 and both first and second‐trimester anomaly scans were reported as normal. A fetal anatomic survey revealed appropriate biometry and the presence of a cystic mass with no solid component measuring 36×29×28 mm located in the anterior mediastinum and extending to the cervical region, apparently tangent to the oesophagus and with a mild displacement of the trachea (Figure 1).

**Figure 2:**
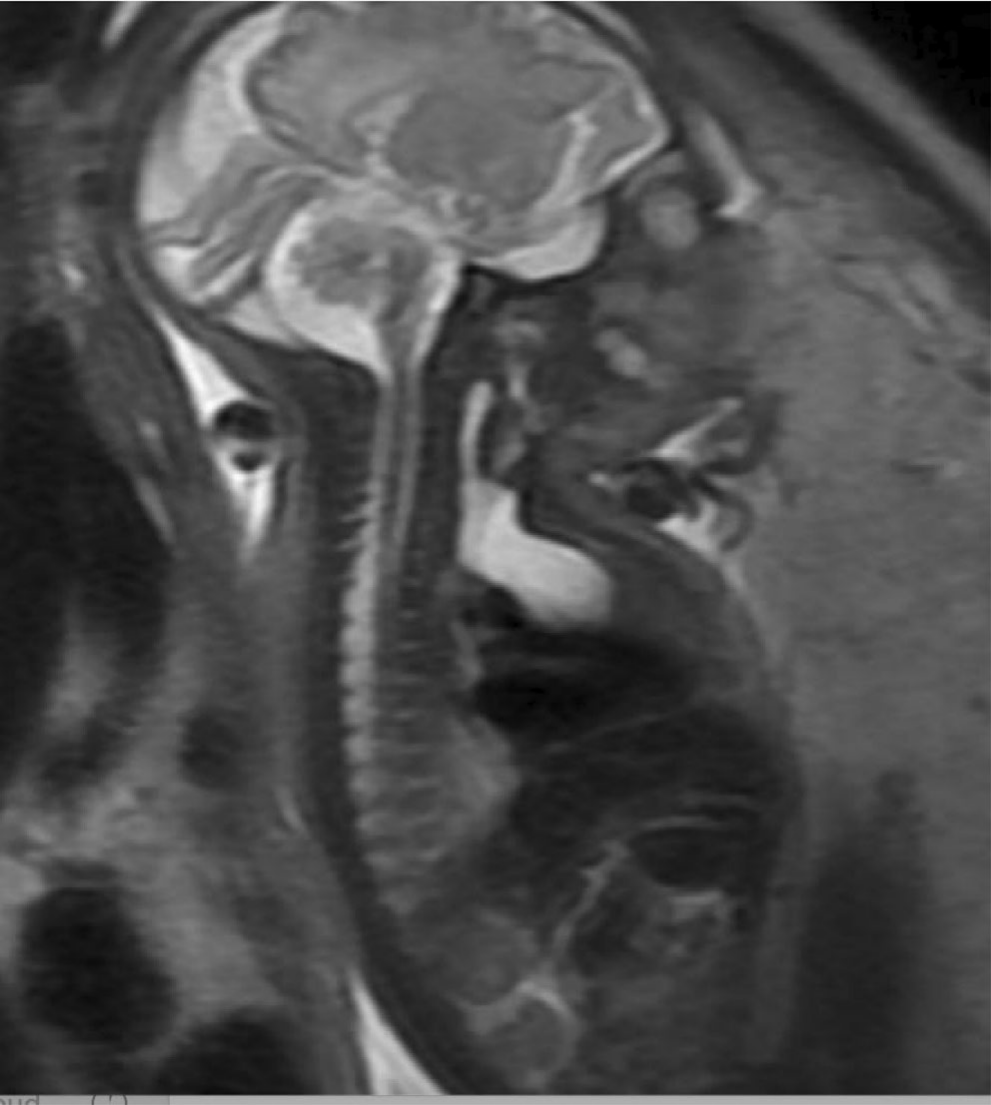
Esophageal duplication cyst at 31 weeks 6 days of pregnancy. Magnetic resonance imaging image showing a fetal cystic mediastinal mass, tangent to the oesophageal wall and communicating with the oesophageal lumen over a distance of 18 mm.

A fetal magnetic resonance imaging (MRI) was performed and the cyst was demonstrated to be a foregut duplication cyst communicating with the oesophageal lumen over a distance of 18 mm (Figure 2).

**Figure 3:**
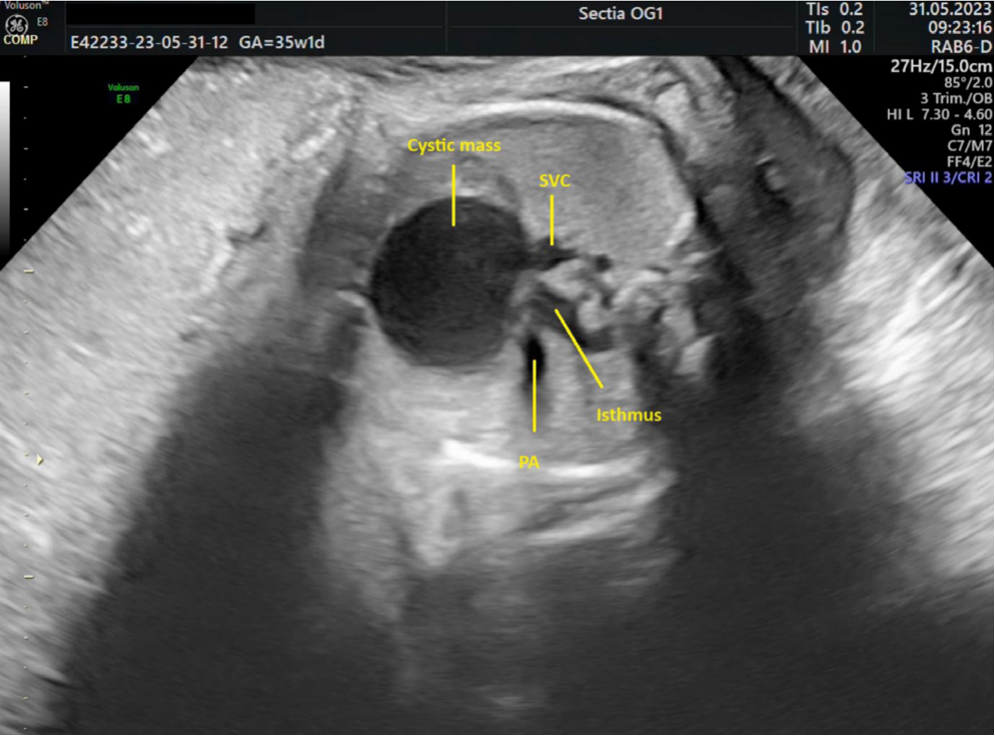
Esophageal duplication cyst at 35 weeks of pregnancy. A transverse view of the chest at the level of the three‐vessel trachea view showed the cystic mass situated close to the aorta without exerting compression on the great vessels.

The transverse view of the chest at the level of the three‐vessel trachea view showed the cystic mass situated close to the aorta, without exerting compression on the great vessels (Figure 3).

**Figure 4:**
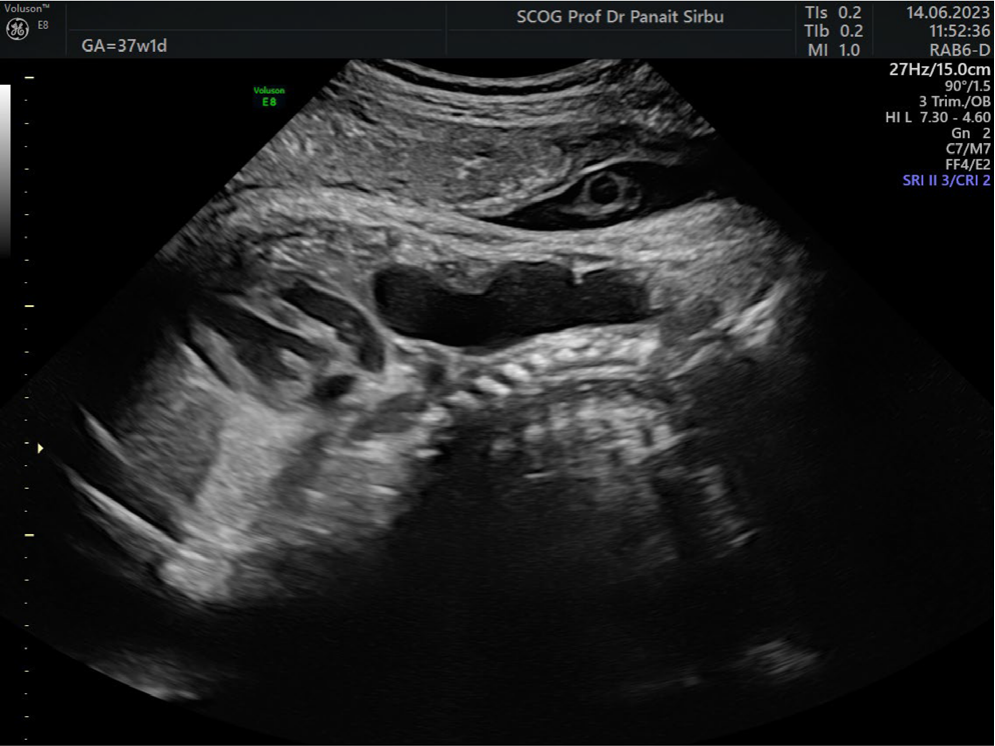
Esophageal duplication cyst at 37 weeks of pregnancy. A sagittal view of the fetal neck and thorax showing the same cystic mass as in Figure 1 with an increased size.

Interestingly, at 37 weeks' gestation ultrasound examination the cyst appeared to be larger, measuring 50×45×23 mm (Figure 4) and presenting peristaltic movements (Video S1).

The patient was delivered at 38 weeks by caesarean section (taking into account the concern for the need for an Ex Utero Intrapartum Treatment (EXIT) procedure) a female newborn weighing 2800 g. Fortunately, the baby had an uneventful post‐natal adaptation. The baby was referred to the paediatric surgery unit where complex imaging studies were performed (oesophagogram, abdominal and thoracic ultrasonography, computed tomography [CT] and MRI and oesophagogastroendoscopy) to confirm the diagnosis. Endoscopic ultrasound‐guided fine‐needle aspiration was not performed in view of high risk of infection. The baby remained completely asymptomatic and gained weight at a satisfactory rate, being exclusively breastfed, the reason for which is the paediatric surgeons decided to defer surgery.

At one month of life, the cystic mass appeared to be larger (120/72/43 mm) and became compressive, and the baby developed severe respiratory distress, so surgical excision was decided. An anterior thoracotomy was performed, and the cyst was completely removed and intact. Fluid content within the cyst was yellowish, and the biochemistry analysis report showed the following: glucose: 48.7 mg/dL, protein: 3.69 g/dL, triglycerides: 104.8 mg/dL and amylase: 211 U/L. Histopathological examination revealed ciliated pseudostratified columnar epithelium with scattered mucus‐secreting cells and two smooth muscle layers in the cyst wall, consistent with tubular ODC.

Post‐operative barium oesophagography showed good peristalsis without any extravasation or stricture. Cervical and thoracic ultrasonographic examinations were normal. The post‐operative course was uneventful, oral feeding was well tolerated, and the baby was discharged after 2 weeks. At the time of writing, the child is eight months old and is growing well without medical complications.

Post‐operative barium oesophagography showed good peristalsis without any extravasation or stricture. Cervical and thoracic ultrasonographic examinations were normal. The post‐operative course was uneventful, oral feeding was well tolerated, and the baby was discharged after 2 weeks. At the time of writing, the child is eight months old and is growing well without medical complications.

Informed consent from the patient regarding the use of information about the diagnosis and also the ultrasonographic and MRI material and evolution of the pregnancy and of the newborn was obtained. The ethics committee from our institution reviewed and approved the publishing of the present manuscript (no.: 19/12/05/2023).

## Discussion

In order to be classified as an oesophageal cyst, a mediastinal cyst must have a close proximity with the oesophagus, be lined by alimentary (squamous epithelium) or tracheobronchial mucosa and be covered by two smooth muscle layers, which is essential to differentiate it from bronchogenic cysts that contain cartilage in their wall.^7^ In our case, the pathology report confirmed the diagnosis and also the communication between the cyst and the native oesophagus lumen (which is reported in only 10% of ODCs).^8^ Moreover, there are three forms of ODC: cystic, tubular and diverticular, with the cystic being the most common and the tubular variety, the rarest,^9^ stressing the distinctiveness of our case.

Diagnosis of ODC prenatally can be challenging because of its rarity and difficult differential diagnosis, as mediastinal masses may have a similar appearance on imagistic studies. It has to be distinguished from bronchogenic cysts (which was our main concern), mature cystic teratomas, pericardial cysts, congenital cystic adenomatoid malformations and neurogenic tumours.^10^ As MRI is the most sensitive and relatively precise tool to make the diagnosis, we used it in order to better characterise the cyst, its communication with the oesophagus, the absence of a concomitant tracheoesophageal fistula and to assess the tracheal deviation. Instead, one weak point of our report is that the prenatal diagnosis was established late during the course of pregnancy, not allowing also to have a better view of the imagistic evolution of the lesion.

Once diagnosis has been confirmed, surgical excision is considered the most appropriate therapeutic option, given the risk for future malignant transformation; the surgical approach can be open, through a laparotomy or thoracotomy, or via minimally invasive techniques dependent on the skill of the surgeon.^7^

## Conclusion

Considering the paucity of the prenatally diagnosed ODC cases published in the English language literature, we are of the opinion that by adding this new case to the ones reported we can expand our knowledge as clinicians on the imagistic features of this entity, thus offering the parents adequate counselling regarding the prognosis and the need for a further multidisciplinary approach after birth.

## Authorship statement

The authorship listing conforms to the journal's authorship policy. All authors are in agreement with the content of the submitted manuscript.

## Funding

No funding information is provided.

## Conflict of interest statement

The authors declare no conflict of interest.

## Supporting information


**Video S1.** Oesophageal duplication cyst at 37 weeks.
